# *HTRA1* Variants and the Interaction with Smoking Confer the Genetic Susceptibility to Ischemic Stroke

**DOI:** 10.7150/ijms.45856

**Published:** 2021-02-23

**Authors:** Yuanrui Tian, Wuzhuang Tang, Song Yang, Yanping Zhao, Yanchun Chen, Xianghai Zhao, Chunlan Liu, Xiaotian Chen, Chong Shen

**Affiliations:** 1 Department of Epidemiology, School of Public Health, Nanjing Medical University, Nanjing 211166, China.; 2 Department of Neurology, Affiliated Yixing People's Hospital of Jiangsu University, People's Hospital of Yixing City, Yixing 214200, China.; 3 Department of Cardiology, Affiliated Yixing People's Hospital of Jiangsu University, People's Hospital of Yixing City, Yixing 214200, China.

**Keywords:** *HTRA1*, ischemic stroke, smoking, interaction, mRNA.

## Abstract

High temperature requirement protein A1 (*HtrA*1) was identified as the causative gene of autosomal recessive arteriopathy and associated with lacunar ischemic stroke (IS) in European. This study aimed at evaluating the association of *HTRA1* with IS and four tagging single-nucleotide polymorphisms (SNPs) were genotyped in a cohort of 4,098 Chinese. The mRNA level of *HTRA1* in 72 IS cases and 72 hypertension controls were measured and compared. In whole population, SNP rs2268350 (C>T) was significantly associated with IS incidence (*P*=0.034). Stratification analysis observed significant association of rs2268350 in male, smoking and drinking populations, rs2672587 (C>G) in smoking and nonsmoking populations and rs3793917 (C>G) in smoking, nonsmoking and nondrinking populations with stroke respectively (*P*<0.05). The additive interaction and multiplicative interaction between rs2268350 and smoking were both of significant (P<0.05) after adjustment for the covariates. There was a cumulated risk of IS among genotypes of rs3793917 (*P*=0.009) and rs2672587 (*P*=0.047) in smoking population. The mRNA level of *HTRA1* in non-smokers with rs2268350 CC was significantly higher than smokers with rs2268350 CT/TT (P=0.046) in IS cases. Our findings support that *HTRA1* confers the genetic susceptibility to IS and smoking might modify the genetic effect of *HTRA1* on IS by suppressing *HTRA1* mRNA expression.

## Introduction

The Global Burden of Disease 2016 Study (GBD 2016) has pointed out that 5.53 million people had different types of strokes worldwide in 2016 1.

Stroke has become the second cause of death and major cause of disability in the world 2 while it's the leading cause of death in China 3. In China, the remarkably increased number of ischemic stroke (IS) has resulted in 301 million disability-adjusted life-years 4.

Stroke including either ischemic or haemorrhagic stroke and about 80% of all strokes are ischemic 5. The predisposing risk factors for stroke include hypertension (HTN), dyslipidemia, impaired glucose metabolism, metabolic obesity, smoking, lack of exercise and a family history of stroke 6, 7. In the pathophysiology of IS, the hazards of smoking on the risk of IS may partly be explained by changes in the venules 8, particularly, wider retinal venular caliber 9. The number of cigarettes smoked daily show a strong dose-response relationship to the incidence of IS among young men 10, as well as, exposure to second-hand smoke can also significantly increase the risk of stroke 11.

The HtrA serine protease 1 (*HTRA1*) was the first one of the serine proteases family to be found 12, 13. *HTRA1* represents diverse biological functions of vascular smooth muscle cell including growth, proliferation, migration, and apoptosis 14, 15. *HTRA1* inhibits transforming growth factor-β (TGF-β) signal transduction by binding TGF-β1, TGF-β2, BMP2, BMP4, activin and Gdf5, thus inducing vascular changes 16-18. In addition, *HTRA1*-mediated proteolysis has been implicated in carcinogenesis 19, age-related macular degeneration (AMD) 20 and familial ischemic cerebral small-vessel disease 21. The *HTRA1*-smoking additive effect was observed on AMD 22 and thus, the interaction between *HTRA1* and smoking on cerebrovascular diseases is very noteworthy.

Hara et al have indicated that *HtrA1* is the causative gene of Cerebral autosomal recessive arteriopathy with subcortical infarcts and leukoencephalopathy (CARASIL), a genetic cause of stroke in the young 21. Recently, two rare monogenic variants were identified by target gene sequencing for younger onset lacunar stroke 23. Patients with CARASIL are characterized by ischemic, non-hypertensive with associated alopecia and spondylosis 24-26. A recent genome-wide association study (GWAS) 27 suggested that a single nucleotide polymorphisms (SNPs) rs79043147 in *HTRA1* was associated with lacunar IS in individuals of European ancestry. However, the minor allele frequency (MAF) of SNP rs79043147 (C>T) is zero in Chinese population. Herein, it is necessary to evaluate whether *HTRA1* harbor susceptible loci for IS in Chinese population.

This study focuses on the association of four tagging SNPs (tagSNPs) at *HTRA1* with the incidence of IS in a cohort study of Chinese Han population and a population-based mRNA levels analysis which would help us well understand the effect of *HTRA1* on IS.

## Method

### Study participants

A cluster sampling method was used to survey 4,128 subjects in 2009, Yixing city and 4,098 individuals without stroke were further followed up from 2014 to 2017. In addition, the mRNA was isolated from peripheral blood mononuclear cells (PBMCs) and compared between 72 IS patients from People's Hospital of Yixing City and age- (±2 years) and gender-matched 72 HTN controls from a community survey. IS cases included in mRNA analysis were diagnosed by CT and/or MRI examination that showed presence of infarction and categorized according to ICD-10 I60-I69.

The trained staff conducted a questionnaire survey to collect demographic data including age, gender, the habits of smoking and drinking and history of disease. The individuals who smoked more than 20 cigarettes per week for at least 3 months per year were defined as smokers. Drinking referred to the habit of currently or previously consuming alcoholic beverages more than 1 time per week for more than 5 months per year. Furthermore, weight (kg) and height (cm) were measured twice and the blood pressure level was averaged by the three times measurements 28.

All the subjects approved to donate 5 ml venous blood. The serum glucose (GLU), total cholesterol (TC), triglyceride (TG), high-density lipoprotein cholesterol (HDL-C) and low-density lipoprotein cholesterol (LDL-C) levels were measured.

During the follow-up study, face to face interview and telephone interview were conducted to determine participants' disease incidence and vital data. The incident events were confirmed by the registered disease and verbal autopsy from the local public health authority (ICD-10 I60-I69) and IS events were finally determined by the study-wide endpoint assessment committee.

The study protocol was approved by the ethics committee of Nanjing Medical University (200803307, 2015077) and each participant signed a written informed consent.

### SNP Selection

In the Han Chinese in Beijing (CHB), China, we searched all 84 SNPs with MAF over 0.05 covered *HTRA1* gene with the addition of 2 kb upstream and 1 kb downstream to screen susceptible SNPs for IS. The tagSNPs were selected according to the criterion of linkage disequilibrium (LD) r²≥0.8, with the data from SNPinfo Web Server (http://snpinfo.niehs.nih.gov/). Finally, from 11 tagSNPs clusters and 16 single SNPs, we selected four tagSNPs in *HTRA1*, rs2268350 (C>T), rs2672587 (C>G), rs3793917 (C>G) and rs12413729 (G>A) with predicted biological function (Supplementary Table S1) that could tag 38 SNPs.

### DNA isolating and SNP genotyping

A standard phenol-chloroform method was used to isolate the genomic DNA. The polymerase chain reaction (PCR)-TaqMan MGB probe array was performed to amplify all four tagSNPs of* HTRA1* by GeneAmp® PCR system 9700 (Applied Biosystems, USA) thermal cycler and then read on the ABI 7900 system (Applied BioSystems, Foster City, CA). All the successful call rates of the four tagSNPs were over 99.79 %.

### RNA Extraction and Real-time PCR detection

After fasting over 12 hours, EDTA-containing blood samples of IS case were collected within 24 hours after admission in hospital and the samples of control were collected during the survey. Anticoagulant samples were immediately mixed with blood preservation solution (Eaglink Cat#EGEN2026, NANJING YININGFUSHENG Biotech. Co., Ltd. Nanjing, China) by 1:3. The total RNA in PBMCs was isolated from 800 µl mixture using RNA Blood Kit (Cat#Yu-B02-1, Yuan Corp., Wuxi, China). cDNA was synthesized from mRNA using TAKARA reverse transcription kits (RR047A Takara PrimeScript RT reagent Kit with gDNA Eraser, Japan). The RT-PCR reactions were performed by ABI RT-PCR 7900. Each sample was prepared with three parallel samples and quality control requires that the standard deviation of cycle threshold (CT) among repeated samples is less than 0.5. The housekeeper gene of Glyceraldehyde-3-phosphate dehydrogenase (GAPDH) was used as reference gene for internal control. The mRNA relative expression is calculated by 2^-∆∆CT^ (∆∆CT case = ∆CT case - ∆CT control average value, ∆∆CT control = ∆CT control - ∆CT control average value, ∆CT = CT of target gene - CT of housekeeper gene). All the reverse transcription and RT-PCR reactions as well as conditions are described at the Supplementary Information Reverse transcription reactions and conditions and RT-PCR reactions and conditions.

### Statistical analysis

The questionnaire data was input by EpiData 3.0 software, IBM-SPSS 20.0was used for the statistical analysis. Cox regression model was used to estimate the association with hazard ratios (HRs) and 95% confidence interval (CI). Mann-Whitney U test was applied to compare the mRNA levels with abnormal distribution between IS cases and controls. Statistical significance was defined as a two-tailed* P* value less than 0.05. False discovery rate (FDR) method performed on R-software (V4.01) was used to correct multiple comparison 29. Moreover, additive interaction was estimated by relative excess risk owing to interaction (RERI), attributable proportion (AP) owing to interaction and synergy index (S). An Excel sheet was used to calculate the additive interaction and their confidence intervals (www.epinet.se).

## Results

### Demographic and clinical characteristics of study population

The participants' demographic and clinical characteristics are listed in Table 1. During the 5.01 years median follow-up period, a total of 187 IS were finally observed by disease register and report system with an incidence density of 88.49 per 10^4^ person-years. Among the 187 IS, 12 IS (6.4%) were less than 55 years old and 175 IS (93.6%) were older than 55 years old. In addition, these 187 IS subjects consist of 94 men (50.3%) and 92 women (49.7%).

### Association analyses of *HTRA1* and IS incidence

Cox regression analysis showed that the rs2268350 (C>T) TT genotype carriers had an increased risk of IS than CC and CT genotype carriers with a marginal *P* value of 0.078, the adjusted HR (95%CI) was 1.499 (1.030-2.181) and *P* value as 0.034 (Supplementary Table S2). Further stratification analysis revealed that in the male and smoking populations, the adjusted HRs (95%CI) were 2.057 (1.240-3.411) and 2.933 (1.464-5.875) with *P* values of 0.005 and 0.002 respectively. In smoking populations, the FDR-adjusted *P* value was 0.048. In drinking population, the genetic variants of rs2268350 presented an additive effect on the risk of IS and the HR (95%CI) after adjustment was 1.855 (1.119-3.074) and *P* value was 0.017 (Table 2).

Moreover, in the non-smoking population, the rs2672587 C>G variation was significantly associated with the increased risk of IS after adjustment for covariates, and HR (95%CI) of additive model was 1.662 (1.076-2.569), *P* = 0.022. While in smoking population, the variants of rs2672587 presented an additive effect on the decreased risk of IS and the adjusted HR (95%CI) was 0.631 (0.405-0.985) with *P* value of 0.043 (Table 2).

In the non-smoking population, rs3793917 CG/GG carriers had an increased risk of IS than CC carriers, the adjusted HR (95%CI) was 2.080 (1.366-3.167), *P* = 0.001. The FDR-adjusted *P* value was 0.048. While in the smoking population, the variants of rs3793917 presented a significant additive effect on the decreased risk of IS, the adjusted HRs (95%CIs) were 0.452 (0.244-0.837) with *P* value of 0.004 (Table 2). The association of rs3793917 (CG/GG vs. CC) with IS was also significant in non-drinking population [adjusted HR (95% CI) =1.647 (1.120-2.420), *P*=0.011] whereas not significant in drinking population (Supplementary Table S3).

### Interaction analysis

Additive interaction analysis showed that additive interaction between *HTRA1* rs2268350 and smoking was statistically significant, after adjustment for age, gender, TC, TG, HDL, LDL, drinking, BMI, T2DM and HTN. The RERI, AP and S values (95% CIs) were 0.271 (-0.071-0.613), 0.169 (0.030-0.308), 1.805 (1.009-3.229) and *P* values were 0.120, 0.017 and 0.047 respectively (Table 3). All the data of other interaction analysis were listed in Supplementary Table S4.

Furthermore, the multiplicative interaction rs2268350 and smoking was also verified to be significant and adjusted HR (95%CI) was 1.179 (1.013-1.372) with *P* of 0.033 after adjustment for covariates.

### Modification effect analysis

For the rs2268350 CC genotype carriers, drinkers had a decreased risk of ischemic stroke than non-drinkers, the HR (95%CI) was 0.285 (0.110-0.734) and *P* value was 0.009, after adjustment for age, gender, TC, TG, HDL, LDL, smoking, BMI, T2DM and HTN. The *P* value of heterogeneity test was 0.005 after adjustments for covariates. For the rs2268350 TT genotype, females had an increased risk of ischemic stroke than males after adjustment for other confounding factors, the adjusted HR (95%CI) was 0.379 (0.144-0.998) and *P* value was 0.049.

According to those who carried CC genotype of rs3793917, smokers had an increased risk of ischemic stroke than CG and GG genotype carriers after adjustment for the other confounding factors, the adjusted HR (95%CI) was 7.503 (2.624-21.457) and *P* value was 0.171×10^-3^. The *P* value for heterogeneity test was less than 0.001 after adjustments for covariates among smokers carrying CC, CG and GG genotypes of rs3793917 (Table 3).

As for carriers with GG genotype of rs2672587, smokers had an increased risk of ischemic stroke than CC and CG genotype carriers, after adjustment for the remaining confounding factors, the adjusted HR (95%CI) was 4.821 (1.526-15.229) with *P* value of 0.007. The *P* value for heterogeneity test was 0.017 after adjustments for covariates among smokers carrying CC, CG and GG genotypes of rs2672587 (Table 4).

### mRNA expression levels comparing between IS cases and controls

The demographic and clinical characteristics for the case-control study of ischemic stroke were listed in Supplementary Table S5. The mRNA expression levels (2^-∆∆CT^) were 1.344 (0.759, 2.452) and 1.112 (0.439, 2.379) in IS cases and controls respectively while no statistical significance was detected (Figure S1), Z=0.944, P=0.345.

In the whole sample of IS cases and controls, smokers had a relative lower *HTRA1* mRNA levels of 0.966 (0.451, 1.805) compared with non-smokers 1.402 (0.609, 2.498) and the *P* value was 0.049. Whereas, there was not the similar difference in IS cases or controls separately.

**Figure 1:**
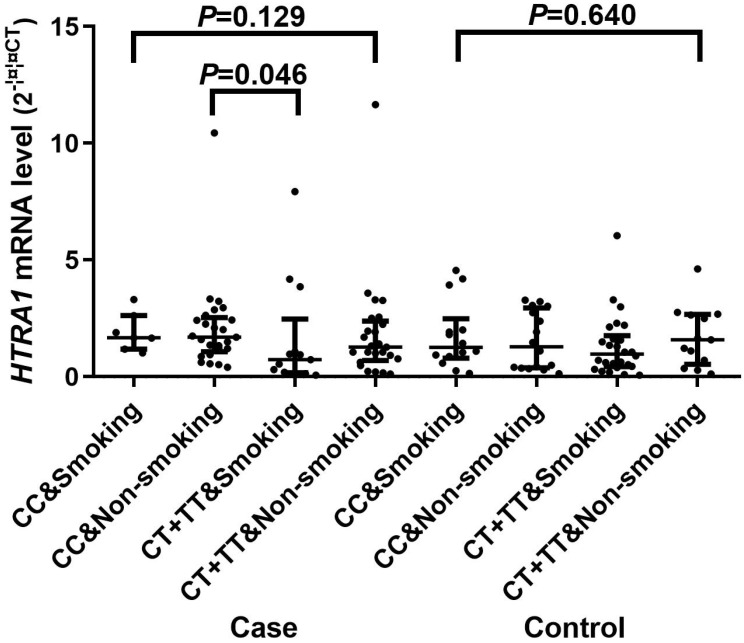
Comparisons of *HTRA1* mRNA levels among smokers with rs2268350 CC, non-smokers with rs2268350 CC, smokers with rs2268350 CT/TT and non-smokers with rs2268350 CT/TT in IS case group and control group respectively.

A significant difference of the mRNA expression levels (2^-∆∆CT^) was found between CC [1.590 (0.945, 2.481)] and CT/TT [0.997 (0.375, 1.982)] genotypes of rs2268350 in IS cases with *P* value of 0.044 but not in controls (*P*>0.05). In addition, the mRNA level (2^-∆∆CT^) of HTRA1 in non-smokers with rs2268350 CC [1.647 (1.137, 2.493)] was significantly higher than smokers with rs2268350 CT/TT [0.686 (0.160, 1.676)] (P=0.046) in IS cases (Figure 1). All the mRNA levels (2^-∆∆CT^) of different genotypes for each tagSNPs were listed in Supplementary Table S6.

## Discussion

In this prospective cohort study of Chinese population, we verified the association of *HtrA1* and IS and identified significant interaction of rs2268350 (C>T) and smoking as well as differential mRNA expression level in PBMCs in IS cases and HTN controls. Notably, both the additive and multiplicative interaction between *HTRA1* rs2268350 and smoking were to be found statistically significant, and the interaction might induce the development of IS probably through suppressing the *HTRA1* mRNA expression in PBMCs. In addition, smoking devotes a two-fold increased risk of rs2672587 and rs3793917 CC genotypes on IS and even more after adjustment for covariates. These findings would further deepen our understanding of the role of gene-environmental interaction in the molecular mechanism of IS.

Cigarette smoke, which contains numerous potential oxidants is associated with the increased blood pressure level and cardiovascular disease mortality 30. Vitro and in vivo studies indicated that the cigarette smoke components exerted their deleterious effects on DNA adducts via cytochrome P-450 (CYP)-dependent bioactivation 31 and inducing oxidative stress 32 and inflammation 33. China now consumes about 40% of the world's total cigarettes consumption, especially in men 34. Previous study has suggested that smoking could strengthen the *HTRA1* additive effect on AMD 22. Similar macular degeneration and cell apoptosis were also observed in patients with neovascular type (nAMD) 35, as well as stroke 36 and myocardial infarction (MI) 37. Cigarette smoking also could modify the genetic effect of LOC387715 rs110490924 on polypoidal choroidal vasculopathy 38. In addition, previous cohort study showed that the nAMD significantly increased the risk of stroke (HR=1.3), particularly hemorrhagic stroke (HR=1.70)^39^. These findings would help to understand the molecular mechanisms how smoking affect stroke and promote tobacco control strategy in preventing the onset of stroke.

For CARASIL, the pathogenic mutations of *HTRA1* were suggested to impair the regulatory function of HTRA1 and thus directly increase the level of TGF-β1 in cerebral small arteries 21. Accordingly, as a downstream effector of the TGF-β1 signaling cascade in brain tissue, the phosphorylated SMAD protein expression levels significantly increased 18. *HTRA1* gene also regulates scar formation in the pathogenesis of scars through the activation of latent TGF-β1 in keloid fibroblasts 40. All these evidences suggest that *HTRA1* participates in the pathogenesis of stroke by regulating angiogenesis via TGF-β signaling.

Our most notable findings illustrated that the additive and multiplicative interactions between smoking and *HTRA1* rs2268350 were both statistically significant on the incidence of IS. Furthermore, among carriers with CC genotype for rs3793917 and rs2672587, smokers always showed a higher risk of IS than non-smokers. Both smoking and the risk allele T of *HTRA1* rs2268350 could suppress the *HTRA1* mRNA expression. Therefore, smoking and the affective allele of *HTRA1* together could be applied to forecast the risk of stroke. The further replication of this interaction would be warranted in other follow-up populations.

The main advantages of this study are that we firstly verified the association of *HtrA1* with IS and the interactions between smoking and *HTRA1* polymorphisms in a cohort study in Chinese population. Particularly, the results of differential *HTRA1* mRNA expression further provided functional evidence of transcriptional level. This study also has the following limitations: firstly, we just selected and tested four tagSNPs of *HtrA1* with MAF over 0.05 and that might miss some rare SNP as well as the biological effect might be presented by closely linked loci nearby. Secondly, we evaluated the interaction of smoking status at baseline and *HtrA1* genetic variations, whereas we didn't check the change of smoking exposure during follow-up and this will prevent us from doing more accurate analysis instead of conventional analytical methods for cohort study. Lastly, although we adjusted the traditional confounders, the findings of this study warrant to be further validated by other studies refraining from potential selection bias.

In conclusion, the findings of this study support that HTRA1 harbor genetic variations that contribute to the susceptibility of IS in Chinese Han population and the population-based evidence of *HTRA1* rs2268350 variations interacting with smoking, and *HTRA1* mRNA differentially expressing among smoking status and rs2268350 genotypes in the patients with IS suggest that smoking modifies the genetic effect of *HTRA1* on IS. Further investigation would be recommended into the exact mechanism of relevant gene-environment interaction.

## Supplementary Material

Supplementary figures and tables.

## Figures and Tables

**Table 1:** Baseline characteristics of the study population

Characteristics	Group	Value
n		4098
Gender	Male	1661(40.5%)
	Female	2437(59.5%)
Age (year)		59.20(52.75-67.00)
Blood pressure (mmHg)	SBP	134(123-141)
	DBP	82(78-89)
Hypertension (%)		1985(48.4%)
TC (mmol/L)		4.79(4.22-5.45)
TG (mmol/L)		1.32(0.90-2.00)
HDL-C (mmol/L)		1.34(1.14-1.56)
LDL-C (mmol/L)		2.65(2.20-3.11)
GLU (mmol/L)		5.28(4.85-5.80)
T2DM (%)		461(11.2%)
BMI (kg/m^2^)		24.0(21.9, 26.4)
BMI Group (%)	Normal (18.5-23.9)	2047(50.0%)
	Overweight (24-27.9)	1521(37.1%)
	Obesity (>28)	530(13.0%)
Smoking	Yes	995(24.3%)
	No	3103(75.7%)
Drinking	Yes	883(21.5%)
	No	3215(78.5)

**Table 2:** Stratification analyses of *HTRA1* polymorphisms and IS incidence in follow-up study

SNP	Stratum	Genotype	IS(n)	Pearson year(y)	Incident density (/10^4^)	HR (95%CI), *P* value		
						Additive model	Dominant model	Recessive model
rs2268350	Male	CC	30	3425.41	87.58	1.356(0.989-1.858)	1.152(0.733-1.811)	2.057(1.24-3.411)
		CT	44	4058.09	108.43	*P*=0.058	*P*=0.539	*P*=0.005
		TT	20	1156.86	172.88			
	Smoking	CC	14	2097.28	66.75	1.767(1.125-2.778)	1.52(0.781-2.959)	2.933(1.464-5.875)
		CT	21	2374.57	88.44	*P*=0.014	*P*=0.217	*P*=0.002
		TT	12	741.86	161.76			
	Drinking	CC	9	1909.32	47.14	1.855(1.119-3.074)	2.942(1.235-7.007)	1.69(0.684-4.175)
		CT	23	2113.95	108.8	*P*=0.017	*P*=0.015	*P*=0.256
		TT	6	640.57	93.67			
rs2672587	Non-smoking	CC	26	4160.35	62.49	1.149(0.906-1.457)	1.662(1.076-2.569)	0.901(0.599-1.357)
		CG	84	7966.11	105.45	*P*=0.253	*P*=0.022	*P*=0.619
		GG	30	3769.38	79.59			
	Smoking	CC	18	1368.69	131.51	0.631(0.405-0.985)	0.608(0.324-1.141)	0.436(0.171-1.112)
		CG	23	2648.71	86.83	*P*=0.043	*P*=0.121	*P*=0.082
		GG	6	1196.32	50.15			
rs3793917	Non-smoking	CC	28	4785.95	58.5	1.328(1.053-1.675)	2.080(1.366-3.167)	1.049(0.693-1.588)
		CG	83	7890.11	105.19	*P*=0.016	*P*=0.001	*P*=0.819
		GG	29	3207.34	90.42			
	Smoking	CC	21	1590.4	132.04	0.498(0.312-0.796)	0.452(0.244-0.837)	0.302(0.100-0.911)
		CG	21	2623.94	80.03	*P*=0.004	*P*=0.011	*P*=0.033
		GG	5	999.38	50.03			
	Non-drinking	CC	35	4932.98	70.95	1.178(0.94-1.476)	1.647(1.120-2.420)	0.91(0.6-1.381)
		CG	86	8204.34	104.82	*P*=0.154	*P*=0.011	*P*=0.657
		GG	28	3297.3	84.92			

Gender stratification adjusted for age, TCH, TG, LDL-C, LDL-C, BMI, drinking, smoking, HTN and T2DM. Smoking stratification adjusted for age, gender, TCH, TG, LDL-C, HDL-C, BMI, drinking, HTN and T2DM. Drinking stratification adjusted for age, gender, TCH, TG, LDL-C, HDL-C, BMI, smoking, HTN and T2DM.

**Table 3:** Additive interaction analysis of gender, smoking, drinking and *HTRA1* polymorphisms on IS incidence

SNPs	Modified factor	Genotype	Stratum	IS(n)	Adjusted HR (95%CI)	Adjusted *P*	Adjusted RERI	Adjusted AP	Adjusted S
rs2268350	Gender	CC	Male	30	1.00(reference)				
		CC	Female	35	0.739(0.424-1.286)	0.284	0.013(-0.132-0.157)	0.013(-0.132-0.158)	0.661(0.000-5979.913)
		CT+TT	Male	64	1.081(0.691-1.693)	0.733	*P*=0.863	*P*=0.861	*P*=0.929
		CT+TT	Female	58	0.849(0.508-1.419)	0.532			
	Smoking	CC	Non-smoking	51	1.00(reference)				
		CC	Smoking	14	0.904(0.457-1.792)	0.773	0.271(-0.071-0.613)	0.169(0.030-0.308)	1.805(1.009-3.229)
		CT+TT	Non-smoking	89	1.028(0.723-1.461)	0.879	*P*=0.120	*P*=0.017	*P*=0.047
		CT+TT	Smoking	33	1.315(0.778-2.225)	0.307			
	Drinking	CC	Non-drinking	56	1.00(reference)				
		CC	Drinking	9	0.385(0.174-0.855)	0.019	0.064(-0.1517-0.2805)	0.065(-0.135-0.269)	0.015(0.000-1.494*10^122^)
		CT+TT	Non-drinking	93	0.949(0.676-1.331)	0.762	*P*=0.559	*P*=0.526	*P*=0.977
		CT+TT	Drinking	29	0.899(0.536-1.508)	0.686			
rs2672587	Smoking	CC	Non-smoking	26	1.00(reference)				
		CC	Smoking	18	2.361(1.212-4.600)	0.012	-0.061(-0.234-0.111)	-0.057(-0.222-0.108)	0.559(0.0484-6.453)
		CG+GG	Non-smoking	114	1.686(1.092-2.602)	0.018	*P*=0.484	*P*=0.498	*P*=0.641
		CG+GG	Smoking	29	1.463(0.792-2.701)	0.224			
rs3793917	Smoking	CC	Non-smoking	28	1.00(reference)				
		CC	Smoking	21	3.211(1.704-6.051)	3.06×10^-4^	-0.0316(-0.21-0.147)	-0.027(0.182-0.128)	0.849(0.305-2.362)
		CG+GG	Non-smoking	112	2.061(1.355-3.135)	0.001	*P*=0.728	*P*=0.735	*P*=0.754
		CG+GG	Smoking	26	1.456(0.786-2.696)	0.232			
	Drinking	CC	Non-drinking	35	1.00(reference)				
		CC	Drinking	14	1.339(0.680-2.640)	0.398	-0.047(-0.18-0.086)	-0.063(-0.257-0.13)	1.227(0.749-2.008)
		CG+GG	Non-drinking	114	1.608(1.095-2.360)	0.015	*P*=0.487	*P*=0.522	*P*=0.416
		CG+GG	Drinking	24	0.913(0.509-1.639)	0.76			

Gender stratification adjusted for age, TCH, TG, LDL-C, LDL-C, BMI, drinking, smoking, HTN and T2DM. Smoking stratification adjusted for age, gender, TCH, TG, LDL-C, HDL-C, BMI, drinking, HTN and T2DM. Drinking stratification adjusted for age, gender, TCH, TG, LDL-C, HDL-C, BMI, smoking, HTN and T2DM.

**Table 4:** Association analysis for the modification effect of gender, smoking and drinking on IS incidence

SNP	Genotype	Modification factor	IS(n)	HR (95%CI)	*P*	Heterogeneity test^b^	HR (95%CI)^a^	*P*^a^	Heterogeneity test^b^
rs2268350	CC	Smoking	65	0.678(0.366-1.255)	0.216	*P*=0.124	1.043(0.483-2.255)	0.915	*P*=0.347
	CT		88	0.978(0.596-1.606)	0.931		0.943(0.485-1.833)	0.863	
	TT		34	1.819(0.883-3.747)	0.105		2.210(0.831-5.873)	0.112	
	CC	Drinking	65	0.329(0.141-0.768)	0.010	*P*=0.037	0.285(0.110-0.734)	0.009	*P*=0.005
	CT		88	1.177(0.727-1.905)	0.508		1.468(0.811-2.657)	0.205	
	TT		34	0.822(0.337-2.005)	0.667		0.368(0.133-1.019)	0.054	
	CC	Gender	65	0.955(0.576-1.582)	0.858	*P*=0.259	0.547(0.296-1.013)	0.055	*P*=0.105
	CT		88	0.841(0.548-1.291)	0.429		1.091(0.618-1.925)	0.765	
	TT		34	0.475(0.238-0.950)	0.035		0.379(0.144-0.998)	0.049	
rs3793917	CC	Smoking	49	2.355(1.316-4.213)	0.004	*P*=0.002	7.503(2.624-21.457)	0.171×10^-3^	*P*<0.001
	CG		104	0.750(0.463-1.213)	0.241		0.659(0.359-1.211)	0.18	
	GG		34	0.397(0.139-1.135)	0.085		0.547(0.156-1.912)	0.3445	
	CC	Drinking	49	0.974(0.496-1.912)	0.94	*P*=0.446	0.635(0.280-1.440)	0.277	*P*=0.967
	CG		104	0.720(0.432-1.200)	0.207		0.727(0.392-1.347)	0.311	
	GG		34	0.435(0.151-1.253)	0.123		0.689(0.203-2.340)	0.551	
rs2672587	CC	Smoking	44	2.076(1.123-3.837)	0.020	*P*=0.015	4.821(1.526-15.229)	0.007	*P*=0.017
	CG		107	0.819(0.514-1.306)	0.402		0.846(0.464-1.539)	0.583	
	GG		36	0.483(0.187-1.253)	0.135		0.616(0.204-1.860)	0.391	

a. Adjusted for age, gender, TC, TG, HDL, LDL, BMI, drinking, smoking, HTN and T2DM. b. *P* value of the heterogeneity test based on the χ^2^ based on Q test.
