# Supplementary material for: HTRA1 Variants and the Interaction with Smoking Confer the Genetic Susceptibility to Ischemic Stroke
Source: Int J Med Sci. 2021 Feb 23;18(8):1840–7. doi: 10.7150/ijms.45856 (PMC7976583; doi:10.7150/ijms.45856)
Supplement: Supplementary file 1 — Supplementary figures and tables. [file ijmsv18p1840s1.pdf]

Supplementary Information

A Prospective Cohort Study on the association of *HTRA1* and Ischemic Stroke in Chinese Han

Yuanrui Tian[1], Wuzhuang Tang[2], Song Yang[3], Yanping Zhao[3], Yanchun Chen[2], Xianghai Zhao[2], Chunlan Liu[1], Xiaotian Chen[1], Lijun Zhu[4], Zhengmei Fang[4], YingShui Yao[4], Chong Shen[1]

1. Department of Epidemiology, School of Public Health, Nanjing Medical University, Nanjing 211166, China

2. Department of Neurology, Affiliated Yixing People's Hospital of Jiangsu University, People's Hospital of Yixing City, Yixing 214200, China

3.Department of Cardiology, Affiliated Yixing People's Hospital of Jiangsu University, People's Hospital of Yixing City, Yixing 214200, China

4. Department of Epidemiology and Biostatistics, School of Public Health, Wannan Medical College, Wuhu 241001, China

Yuanrui Tian, Wuzhuang Tang and Song Yang equally contributed to this work.

To whom correspondence should be addressed:

Chong Shen, Ph.D., Professor

Department of Epidemiology, School of Public Health, Nanjing Medical University, 101 Longmian Avenue, Jiangning, Nanjing, 211166 China

Tel: +86 25 86868291, Fax: +86 25 86527613, E-mail: sc100@126.com

Methods

Reverse transcription reactions and conditions

The reverse transcription reactions were performed in a 20 μl reaction mixture including 0.3 μg RNA, 2 μl 5'gDNA Eraser Buffer, 1 μl gDNA Eraser，4μl Nuclease-Free Water，4μl 5 x Primescript Buffer 2, 1μl Enzyme Mix1 and 1μl RT Primer Mix. The PCR conditions were 67 ℃ for 15min，85 ℃ for 5s.

RT-PCR reactions and conditions

According to the NCBI Nucleotide database, the human HTRA1 has only 1 transcript variant, the primer sequences were listed as follows: HTRA1, 5-GTCTGGGTTTATTGTGTCGGAA-3 (forward) and 5-CGTTCTTCAGCTCAACTTTGACC-3 (reverse); GAPDH, 5-GCACCGTCAAGGCTGAGAAC-3 (forward) and 5-TGGTGAAGACGCCAGTGGA-3 (reverse). The primers were designed using Primer Bank (https://pga.mgh.harvard.edu/primerbank/) or Primer Premier 3 (http://bioinfo.ut.ee/primer3-0.4.0/). Real-time PCR (RT-PCR) reactions performed in a 10 μl reaction mixture including 2 μl cDNA, 5 μl HieffTM qPCR SYBR GEEN Master Mix, 2.6μl Nuclease-Free water, 0.2 μl upstream primer and 0.2 μl downstream primer. The RT-PCR conditions were 95℃ for 5 min, 95℃ for 10s, 60℃ for 20s and 72℃ for 20s with forty cycles. Melting curve includes 1 cycle with 95°C for 15s, 60°C for 1min and 95°C for 15s. Three parallel samples were set up with a standard deviation less than 0.5, and the average value was calculated.

Supplementary Table 1 Bioinformatics of the four tagSNPs in *HTRA1*

SNP	Allele	Chr	Position	Location	MAF	HWE$^{\alpha}$	TFBS	RegulomeDB
rs2268350	C>T	10	124241050	intronic	0.227	0.548		2a
rs2672587	C>G	10	124225345	intronic	0.464	0.342		2b
rs3793917	C>G	10	124209265	1.8kb from 5' UTR	0.466	0.771	Yes	4
rs12413729	G>A	10	124249120	intronic	0.131	0.444		2b

SNP: single nucleotide polymorphisms; Chr: Chromosome; MAF: minor allele frequency; HWE: Hardy-Weinberg equilibrium; TFBS: Transcription Factor Binding Site.

a. χ^2 test was used to assess HWE in control.

Supplementary Table 2 Association analysis of *HTRA1* polymorphisms and IS incidence

SNP	Genotype	IS (n)	Pearson year(y)	Incident density (10^4)	Model, HR (95%CI)*, P		
					Additive model	Dominant model	Recessive model
rs2268350	CC	65	8306.07	78.26	1.19(0.96-1.475)	1.117(0.821-1.52)	1.499(1.030-2.181)
	CT	88	9901.61	88.87	*P*=0.112	*P*=0.482	*P*=0.034
	TT	34	2896.86	117.37			
rs2672587	CC	44	5529.04	79.58	0.989(0.806-1.214)	1.253(0.885-1.774)	0.755(0.521-1.093)
	CG	106	10614.82	99.86	*P*=0.918	*P*=0.204	*P*=0.137
	GG	37	4965.70	74.51			
rs3793917	CC	49	6376.34	76.85	1.062(0.867-1.301)	1.341(0.962-1.868)	0.829(0.567-1.212)
	CG	104	10514.05	98.92	*P*=0.562	*P*=0.083	*P*=0.333
	GG	34	4206.72	80.82			
rs12413729	GG	148	15885.92	93.16	0.878(0.631-1.221)	0.84(0.587-1.201)	1.296(0.412-4.081)
	GA	36	4874.21	73.86	*P*=0.438	*P*=0.339	*P*=0.658
	AA	3	356.67	84.11			

*Adjusted for age, gender, TC, TG, LDL, HDL, BMI, smoking, drinking, HTN and T2DM

Supplementary Table 3 Stratification analyses for the association between four SNPs and IS incidence

SNP	Group	Genotype	IS(n)	Pearson year(y)	Incident density (10^4)	Model, HR (95%CI)*, *P*		
						Additive model	Dominant model	Recessive model
rs2268350	Male	CC	30	3425.41	87.58	1.356(0.989-1.858)	1.152(0.733-1.811)	2.057(1.24-3.411)
		CT	44	4058.09	108.43	*P*=0.058	*P*=0.539	*P*=0.005
		TT	20	1156.86	172.88			
	Female	CC	35	4880.66	71.71	1.08(0.8-1.458)	1.114(0.725-1.712)	1.091(0.611-1.95)
		CT	44	5843.52	75.30	*P*=0.616	*P*=0.622	*P*=0.768
		TT	14	1740.00	80.46			
rs2268350	＜55	CC	5	3029.5	16.50	1.29(0.532-3.13)	1.128(0.31-4.108)	1.97(0.425-9.129)
		CT	4	3634.7	11.01	*P*=0.573	*P*=0.855	*P*=0.386
		TT	3	1014.7	29.57			
	⩾55	CC	60	5276.6	113.71	1.181(0.946-1.474)	1.128(0.82-1.551)	1.441(0.974-2.132)
		CT	84	6267.0	134.04	*P*=0.142	*P*=0.459	*P*=0.067
		TT	31	1882.2	164.70			
rs2268350	Nonsmoking	CC	51	6208.79	82.14	1.099(0.855-1.411)	1.053(0.74-1.499)	1.277(0.807-2.021)
		CT	67	7527.04	89.01	*P*=0.461	*P*=0.773	*P*=0.297
		TT	22	2155	102.09			
	Smoking	CC	14	2097.28	66.75	1.767(1.125-2.778)	1.52(0.781-2.959)	2.933(1.464-5.875)
		CT	21	2374.57	88.44	*P*=0.014	*P*=0.217	*P*=0.002
		TT	12	741.86	161.76			
rs2268350	Nondrinking	CC	56	6396.76	87.54	1.114(0.876-1.418)	0.977(0.695-1.372)	1.49(0.984-2.255)
		CT	65	7787.67	83.47	*P*=0.379	*P*=0.892	*P*=0.06
		TT	28	2256.29	124.1			
	Drinking	CC	9	1909.32	47.14	1.855(1.119-3.074)	2.942(1.235-7.007)	1.69(0.684-4.175)
		CT	23	2113.95	108.8	*P*=0.017	*P*=0.015	*P*=0.256

		TT	6	640.57	93.67			
rs2672587	Male	CC	24	2244.60	106.92	0.899(0.673-1.202)	1.15(0.706-1.874)	0.616(0.357-1.063)
		CG	53	4306.79	123.06	*P*=0.473	*P*=0.575	*P*=0.082
		GG	17	2088.97	81.38			
	Female	CC	20	3284.44	60.89	1.083(0.808-1.452)	1.357(0.822-2.238)	0.908(0.545-1.511)
		CG	54	6308.03	85.61	*P*=0.592	*P*=0.233	*P*=0.710
		GG	19	2876.73	66.05			
rs2672587	＜55	CC	2	1979.3	10.10	1.131(0.458-2.791)	1.236(0.256-5.959)	1.13(0.278-4.591)
		CG	6	3803.3	15.78	*P*=0.79	*P*=0.792	*P*=0.864
		GG	4	1901.3	21.04			
	⩾55	CC	42	3549.8	118.32	0.971(0.785-1.2)	1.203(0.843-1.715)	0.741(0.504-1.091)
		CG	101	6811.5	148.28	*P*=0.783	*P*=0.309	*P*=0.129
		GG	32	3064.4	104.43			
rs2672587	Nonsmoking	CC	26	4160.35	62.49	1.149(0.906-1.457)	1.662(1.076-2.569)	0.901(0.599-1.357)
		CG	84	7966.11	105.45	*P*=0.253	*P*=0.022	*P*=0.619
		GG	30	3769.38	79.59			
	Smoking	CC	18	1368.69	131.51	0.631(0.405-0.985)	0.608(0.324-1.141)	0.436(0.171-1.112)
		CG	23	2648.71	86.83	*P*=0.043	*P*=0.121	*P*=0.082
		GG	6	1196.32	50.15			
rs2672587	Nondrinking	CC	32	4274.97	74.85	1.022(0.812-1.286)	1.342(0.901-1.999)	0.778(0.516-1.174)
		CG	88	8339.87	105.52	*P*=0.855	*P*=0.148	*P*=0.232
		GG	29	3830.88	75.7			
	Drinking	CC	12	1254.07	95.69	0.854(0.529-1.377)	1.044(0.482-2.261)	0.561(0.219-1.439)
		CG	19	2274.95	83.52	*P*=0.517	*P*=0.913	*P*=0.229
		GG	7	1134.82	61.68			
rs3793917	Male	CC	28	2612.54	107.18	0.85(0.632-1.144)	1.003(0.635-1.586)	0.569(0.313-1.037)

		CG	52	4285.23	121.35	*P*=0.283	*P*=0.989	*P*=0.066
		GG	14	1735.05	80.69			
	Female	CC	21	3763.81	55.79	1.326(0.999-1.76)	1.91(1.156-3.154)	1.135(0.689-1.869)
		CG	52	6228.82	83.48	*P*=0.051	*P*=0.011	*P*=0.618
		GG	20	2471.67	80.92			
rs3793917	＜55	CC	2	2349.8	8.51	1.494(0.607-3.676)	1.942(0.393-9.602)	1.531(0.369-6.346)
		CG	6	3832.9	15.65	*P*=0.382	*P*=0.416	*P*=0.557
		GG	4	1496.3	26.73			
	⩾55	CC	47	4026.5	116.73	1.038(0.842-1.281)	1.297(0.923-1.822)	0.799(0.537-1.19)
		CG	98	6681.2	146.68	*P*=0.725	*P*=0.134	*P*=0.269
		GG	30	2710.4	110.68			
rs3793917	Nonsmoking	CC	28	4785.95	58.5	1.328(1.053-1.675)	2.08(1.366-3.167)	1.049(0.693-1.588)
		CG	83	7890.11	105.19	*P*=0.016	*P*=0.001	*P*=0.819
		GG	29	3207.34	90.42			
	Smoking	CC	21	1590.4	132.04	0.498(0.312-0.796)	0.452(0.244-0.837)	0.302(0.1-0.911)
		CG	21	2623.94	80.03	*P*=0.004	*P*=0.011	*P*=0.033
		GG	5	999.38	50.03			
rs3793917	Nondrinking	CC	35	4932.98	70.95	1.178(0.94-1.476)	1.647(1.12-2.42)	0.91(0.6-1.381)
		CG	86	8204.34	104.82	*P*=0.154	*P*=0.011	*P*=0.657
		GG	28	3297.3	84.92			
	Drinking	CC	14	1443.36	97	0.666(0.398-1.114)	0.671(0.322-1.399)	0.427(0.14-1.301)
		CG	18	2309.71	77.93	*P*=0.121	*P*=0.287	*P*=0.134
		GG	6	909.42	65.98			
rs12413729	Male	GG	74	6503.71	113.78	1.007(0.634-1.598)	0.944(0.569-1.567)	2.025(0.49-8.362)
		GA	18	1989.75	90.46	*P*=0.977	*P*=0.825	*P*=0.330
		AA	2	153.12	130.62			

	Female	GG	74	9382.21	78.87	0.733(0.442-1.216)	0.706(0.41-1.218)	0.799(0.11-5.825)
		GA	18	2884.46	62.40	*P*=0.230	*P*=0.211	*P*=0.825
		AA	1	203.54	49.13			
rs12413729	＜55	GG	10	5760.2	17.36	1.094(0.302-3.963)	0.724(0.135-3.897)	5.613(0.414-76.146)
		GA	1	1804.6	5.54	*P*=0.891	*P*=0.707	*P*=0.195
		AA	1	125.3	79.80			
	⩾55	GG	138	10125.7	136.29	0.877(0.623-1.234)	0.863(0.598-1.248)	0.906(0.223-3.678)
		GA	35	3069.6	114.02	*P*=0.451	*P*=0.434	*P*=0.891
		AA	2	231.4	86.45			
rs12413729	Nonsmoking	GG	114	12029.25	94.77	0.816(0.543-1.225)	0.811(0.526-1.25)	0.653(0.091-4.711)
		GA	25	3610.39	69.24	*P*=0.327	*P*=0.342	*P*=0.673
		AA	1	257.22	38.88			
	Smoking	GG	34	3856.66	88.16	0.989(0.539-1.814)	0.846(0.429-1.667)	3.206(0.748-13.738)
		GA	11	1263.82	87.04	*P*=0.971	*P*=0.628	*P*=0.117
		AA	2	99.45	201.11			
rs12413729	Nondrinking	GG	118	12427.4	94.95	0.86(0.592-1.248)	0.836(0.558-1.252)	1.006(0.248-4.089)
		GA	29	3745.88	77.42	*P*=0.427	*P*=0.385	*P*=0.993
		AA	2	273.46	73.14			
	Drinking	GG	30	3458.52	86.74	1.077(0.512-2.265)	0.973(0.429-2.205)	3.165(0.406-24.676)
		GA	7	1128.33	62.04	*P*=0.845	*P*=0.948	*P*=0.271
		AA	1	83.2	120.19			

Age stratification adjusted for age, gender, TCH, TG, LDL-C, LDL-C, BMI, drinking, smoking, HTN and T2DM.

Gender stratification adjusted for age, TCH, TG, LDL-C, LDL-C, BMI, drinking, smoking, HTN and T2DM.

Smoking stratification adjusted for age, gender, TCH, TG, LDL-C, LDL-C, BMI, drinking, HTN and T2DM.

Drinking stratification adjusted for age, gender, TCH, TG, LDL-C, LDL-C, BMI, smoking, HTN and T2DM.

Supplementary Table 4 Additive interaction of gender, smoking, drinking and HTRA1 polymorphisms on ischemic stroke

SNPs	Modification factor	Genotypes	Stratum	IS(n)	HR (95%CI)	*P*	RERI (95%CI)	AP (95%CI)	S (95%CI)
rs2268350	Gender	CC	Male	30	1.00(reference)				
		CC	Female	35	0.977(0.595-1.605)	0.928	-0.032(-0.162-0.156)	-0.003(-0.161-0.154)	0.778(0.000-1207458.066)
		CT+TT	Male	64	1.414(0.911-2.194)	0.122	*P*=0.969	*P*=0.969	*P*=0.973
		CT+TT	Female	58	1.014(0.648-1.588)	0.951			
	Smoking	CC	Nonsmoking	51	1.00(reference)				
		CC	Smoking	14	0.637(0.345-1.178)	0.150	0.183(-0.035-0.401)	0.137(0.000-0.273)	2.154(1.173-3.955)
		CT+TT	Nonsmoking	89	1.035(0.731-1.464)	0.847	*P*=0.099	*P*=0.050	*P*=0.013
		CT+TT	Smoking	33	1.212(0.782-1.880)	0.390			
	Drinking	CC	Nondrinking	56	1.00(reference)				
		CC	Drinking	9	0.365(0.172-0.773)	0.009	0.065(-0.141-0.270)	0.063(-0.123-0.248)	-0.950
		CT+TT	Nondrinking	93	0.974(0.697-1.362)	0.879	*P*=0.054	*P*=0.054	
		CT+TT	Drinking	29	1.024(0.651-1.612)	0.918			
rs2672587	Smoking	GG	Nonsmoking	26	1.00(reference)				
		GG	Smoking	18	2.024(1.107-3.699)	0.022	-0.816(-0.2-0.037)	-0.1(0.257-0.058)	1.805(0.671-4.855)
		GC+CC	Nonsmoking	114	1.605(1.047-2.460)	0.030	*P*=0.177	*P*=0.214	*P*=0.242
		GC+CC	Smoking	29	1.130(0.661-1.932)	0.656			
rs3793917	Smoking	CC	Nonsmoking	28	1.00(reference)				
		CC	Smoking	21	2.205(1.251-3.887)	0.006	-0.064(-0.194-0.067)	-0.07(-0.225-0.085)	3.355(0.078-145.355)
		CG+GG	Nonsmoking	112	1.787(1.180-2.707)	0.006	*P*=0.341	*P*=0.376	*P*=0.529
		CG+GG	Smoking	26	1.137(0.661-1.954)	0.643			
	Drinking	CC	Nondrinking	35	1.00(reference)				
		CC	Drinking	14	1.144(0.612-2.139)	0.674	-0.053(-0.17-0.064)	-0.073(-0.247-0.102)	1.241(0.815-1.891)
		CG+GG	Nondrinking	114	1.414(0.967-2.068)	0.074	*P*=0.375	*P*=0.414	*P*=0.314
		CG+GG	Drinking	24	0.890(0.524-1.510)	0.665			

Gender stratification adjusted for age, TCH, TG, LDL-C, LDL-C, BMI, drinking, smoking, HTN and T2DM.
Smoking stratification adjusted for age, gender, TCH, TG, LDL-C, LDL-C, BMI, drinking, HTN and T2DM.
Drinking stratification adjusted for age, gender, TCH, TG, LDL-C, LDL-C, BMI, smoking, HTN and T2DM.

Supplementary Table 5 Demographic and clinical characteristics for the case-control study of ischemic stroke

Characteristics	Group	Controls n=72	IS cases n=72	F/χ^2	*P*
Age (year)		62.6±7.9	63.14±8.6	0.392	0.696
Gender (%)	Male	48(66.7%)	48(66.7%)	0	1
	Female	24(33.3%)	24(33.3%)		
Smokers (%)	Yes	42(58.3%)	19(27.5%)	13.614	<0.001
	No	30(41.7%)	50(72.5%)		
Drinkers (%)	Yes	38(52.8%)	9(12.7%)	26.055	<0.001
	No	34(47.2%)	62(87.3%)		
SBP		152.56±21.64	155.3±21.15	0.731	0.466
DBP		83.56±10.70	89.59±12.45	2.977	<0.01
Hypertension (%)	Yes	24(33.3%)	45(72.6%)	20.554	<0.001
	No	48(66.7%)	17(27.4%)		
T2DM (%)	Yes	15(20.8%)	12(19.4%)	0.045	0.832
	No	57(79.2%)	50(80.6%)		

SBP, systolic blood pressure; DBP, diastolic blood pressure; T2DM, type 2 diabetes mellitus; IS: ischemic stroke.

* and ** indicates that the statistical difference when compared with the controls;

*, *P*<0.05; **, *P*<0.001.

Supplementary Table 6 Comparisons of *HTRA1* mRNA ($2^{-\triangle\triangle CT}$) level among different genotypes of four tagSNPs in additive model and dominant model

Group	SNP	Stratum	n	WT	HT	MT	*P*[a]	*P*[b]
Case	rs2268350	All cases	31/33/5	1.70(1.18-2.62)	1.05(0.51-2.10)	1.05(0.19-7.92)	0.116	0.044
	(C>T)	Smoking	7/11/2	1.67(1.18-2.62)	0.73(0.20-1.07)	2.16(0.14-)	0.148	0.067
		Nonsmoking	24/22/3	1.72(1.01-2.59)	1.29(0.73-2.31)	1.05(0.24-)	0.521	0.254
	rs2672587	All cases	15/39/15	1.07(0.52-1.89)	1.69(0.77-2.62)	1.22(0.73-2.96)	0.517	0.282
	(C>G)	Smoking	5/13/2	1.18(1.01-1.77)	1.02(0.17-3.58)	0.85(0.73-)	0.740	0.612
		Nonsmoking	10/26/13	0.99(0.42-2.14)	1.72(1.05-2.47)	1.33(0.78-3.12)	0.345	0.163
	rs3793917	All cases	22/39/8	1.62(0.90-2.54)	1.20(0.54-2.43)	1.50(1.09-2.83)	0.446	0.503
	(C>G)	Smoking	6/13/1	1.77(1.15-3.01)	0.73(0.17-2.16)	1.67	0.11	0.062
		Nonsmoking	16/26/7	1.48(0.68-2.44)	1.55(0.73-2.47)	1.33(1.05-2.96）	0.785	0.685
	rs12413729	All cases	54/12/3	1.32(0.70-2.30)	2.13(0.98-3.72)	1.07(0.21-)	0.364	0.415
	(G>A)	Smoking	14/4/2	0.98(0.28-1.90)	2.42(0.29-6.92)	1.48(1.07-)	0.65	0.397
		Nonsmoking	40/8/1	1.38(0.88-2.38)	2.13(1.02-3.18)	0.21	0.184	0.657
Control	rs2268350	All controls	30/24/15	1.27(0.42-2.81)	1.32(0.47-2.25)	0.71(0.32-2.20)	0.651	0.579
	(C>T)	Smoking	14/17/9	1.26(0.80-2.49)	1.29 (0.51-1.88)	0.61(0.28-1.63)	0.352	0.292
		Nonsmoking	16/7/6	1.29(0.38-2.95)	1.65(0.35-2.76)	1.35(0.60-2.65)	0.946	0.948
	rs2672587	All controls	16/25/27	1.96(0.69-2.69)	1.09(0.45-2.03)	0.89(0.40-2.29)	0.379	0.165
	(C>G)	Smoking	10/16/13	1.86(0.56-2.70)	1.07(0.59-1.96)	0.71(0.42-1.59)	0.377	0.258
		Nonsmoking	6/9/14	2.44(0.86-2.83)	1.11(0.38-2.28)	1.34(0.36-3.09)	0.691	0.546
	rs3793917	All controls	16/35/18	1.62(0.42-2.47)	0.94(0.41-2.01)	1.48(0.59-3.00)	0.391	0.722
	(C>G)	Smoking	10/20/10	0.96(0.23-1.92)	0.99(0.58-2.10)	1.32(0.59-1.97)	0.663	0.414
		Nonsmoking	6/15/8	2.44(1.62-2.83)	0.49(0.34-1.65)	2.14(0.64-3.17)	0.092	0.142
	rs12413729	All controls	51/14/5	1.10(0.41-2.38)	1.28(0.64-2.28)	0.71(0.38-1.73)	0.598	0.963

(G>A)	Smoking	32/5/4	1.07(0.53-2.10)	1.34(0.75-1.91)	0.66(0.26-0.76)	0.253	0.466
	Nonsmoking	19/9/1	1.47(0.35-2.76)	1.22(0.54-2.57)	2.68	0.701	0.769

WT: wild type; HT: heterozygote; MT: mutant type.

a. *P* for additive model

b. *P* for dominant model

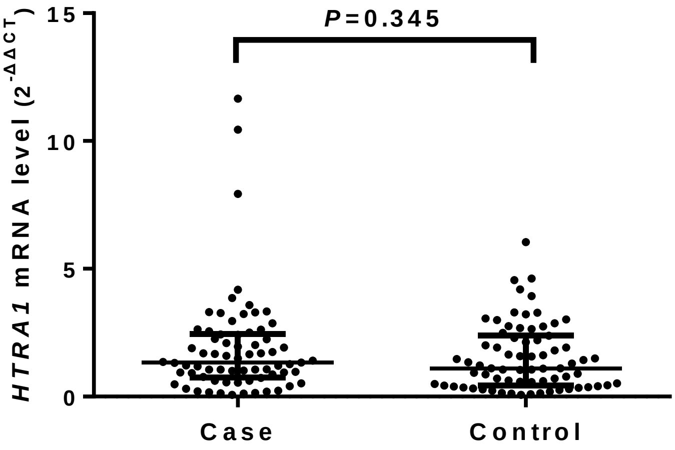

e-Figure 1. Comparison of *HTRA1* mRNA levels between IS case group and control group.
